# Draft Genome Sequence of an NDM-1-Producing Sequence Type 101 (ST101) Klebsiella pneumoniae Strain, Marseille-Q1949

**DOI:** 10.1128/MRA.00437-21

**Published:** 2021-07-08

**Authors:** Amanda Chamieh, Rita Zgheib, Sabah El-Sawalhi, Eid Azar, Jean-Marc Rolain

**Affiliations:** a Division of Infectious Diseases, Saint George Hospital University Medical Center, Beirut, Lebanon; b Aix-Marseille Université, Institut de Recherche pour le Développement (IRD), UMR Microbes Evolution Phylogeny and Infections (MEPHI), Marseille, France; c Institut Hospitalo-Universitaire Méditerranée Infection, Marseille, France; d Aix Marseille Université, Institut de Recherche pour le Développement (IRD), Service de Santé des Armées, AP-HM, UMR Vecteurs Infections Tropicales et Méditerranéennes (VITROME), Marseille, France; Loyola University Chicago

## Abstract

A pan-drug-resistant Klebsiella pneumoniae strain was isolated from the blood of a 70-year-old critically ill patient in April 2019. Interestingly, the patient recovered and was discharged home a month later. The genome of strain Marseille-Q1949 is 5,607,584 bp long and has a 57.1% G+C content and 5,467 protein-coding genes.

## ANNOUNCEMENT

On 12 April 2019, we isolated and sequenced a pan-drug-resistant Klebsiella pneumoniae strain from the blood of a 70-year-old patient who had been in the intensive care unit since March 2019. The patient was discharged home in June 2019 despite complications. At Saint George Hospital University Medical Center (SGHUMC), Infection Control has a constitutional institutional review board (IRB) waiver to retrieve relevant records of patients with pathogens of high importance.

Growth was detected after 24 h of inoculation of 10 ml blood into Bactec Plus Aerobic/F vials and incubation in Bactec 9240 at 35°C. The isolate was inoculated on MacConkey agar, incubated at 37°C for 24 h, and identified as K. pneumoniae by matrix-assisted laser desorption ionization–time of flight mass spectrometry (MALDI-TOF MS).

Antimicrobial susceptibility testing was performed on Mueller-Hinton agar using the disc-diffusion method per EUCAST 2020. Etest was additionally performed for ertapenem and imipenem (https://www.eucast.org/clinical_breakpoints/). Colistin susceptibility was tested with the UMIC method (1). Among the tested antimicrobials, strain Marseille-Q1949 was only susceptible to fosfomycin and doxycycline.

Genomic DNA (gDNA) of strain Marseille-Q1949 was extracted on the EZ1 BioRobot system using an EZ1 DNA tissue kit (Qiagen, Germany) and quantified using a Qubit assay with a high-sensitivity kit (Life Technologies, Carlsbad, CA, USA) to 0.2 ng/μl. Genomic DNA was sequenced on the MiSeq platform (Illumina Inc., San Diego, CA, USA) with the paired-end strategy. The library was prepared following the workflow of the Nextera XT DNA library prep kit (Illumina) (2). Automated cluster generation and paired-end sequencing with dual index reads were performed in a single 39-h run in 2 × 250 bp. We obtained a total of 3.8 Gb of information from a 416,000/mm^2^ cluster density with a cluster passing quality control filter of 91.7%. Within this run, the index representation for K. pneumoniae strain Marseille-Q1949 was 3.87%. Using FastQC v0.11.8 (https://www.bioinformatics.babraham.ac.uk/projects/fastqc/), we filtered the 7,995,112 paired-end reads obtained for the complete run and 284,204 reads for the genome of strain Marseille-Q1949. Finally, the forward (39,812 kb) and reverse (41,825 kb) strands were assembled using SPAdes v3.13 as previously described (3, 4). K. pneumoniae strain Marseille-Q1949 has a 5,607,584-bp-long genome with a G+C content of 57.1%. The genome was assembled into 274 contigs (*N*_50_, 40,381 bp; *L*_50_, 42) with a coverage of 12.69×. The 16S rRNA gene sequence was extracted from the genome using Barrnap v0.9 (https://github.com/tseemann/barrnap). Using BLASTn against the NCBI 16S rRNA database, we confirmed that strain Marseille-Q1949 belongs to K. pneumoniae. The genome was annotated online using the NCBI Prokaryotic Genome Annotation Pipeline (PGAP) v5.1. We obtained 5,641 coding genes, 5,467 of which were protein-coding genes. A total of 55 coding genes were annotated as RNAs—6 rRNAs, 41 tRNAs, and 8 noncoding RNAs (ncRNAs). The remaining 119 coding genes were pseudogenes. Multilocus sequence typing (MLST) analysis using the MLST tool v2.19.0 (https://github.com/tseemann/mlst) (5) assigned sequence type 101 (ST101) to strain Marseille-Q1949.

Default parameters were used for all software tools.

ResFinder v4.1 identified the following antimicrobial resistance genes with a 90% identity threshold (6): *aac(3)-IIa*, *aac(6′)-Ib*, *aac(6′)-Ib-cr*, *aadA1*, *aph(3′)-VI*, *aph(6)-Id*, *bla*_CTX-M-15_, *bla*_NDM-1_, *bla*_OXA-1_, *bla*_OXA-9_, *bla*_SHV-28_, *bla*_SHV-106_, *bla*_TEM-1C_, *catB4*, *dfrA14*, *qnrS1*, and *strA*. PlasmidFinder v2.1 with a 95% identity threshold (7) identified plasmids IncFIB(K), IncFIB(pQil), and IncR.

### Data availability.

The draft genome and read sequences of K. pneumoniae strain Marseille-Q1949 (BioProject PRJNA697840; BioSample SAMN17673831) have been deposited at GenBank under accession numbers JAFEVI000000000 and SRR13576949, respectively. The genome version described here is JAFEVI010000000.
